# Bilateral medialization thyroplasty in patients with vocal fold atrophy with or without sulcus

**DOI:** 10.1007/s00405-020-05933-9

**Published:** 2020-04-02

**Authors:** Emke M. J. M. van den Broek, Bas J. Heijnen, Martine Hendriksma, Vivienne A. H. van de Kamp-Lam, Thijs O. Verhagen, Antonius P. M. Langeveld, Peter Paul G. van Benthem, Elisabeth V. Sjögren

**Affiliations:** 1 Department of Otorhinolaryngology/Head and Neck Surgery, Leiden University Medical Center, Albinusdreef 2, PO-Box 9600, 2300 RC Leiden, The Netherlands; 2 Department of Otorhinolaryngology/Head and Neck Surgery, University Medical Center, Utrecht, The Netherlands

**Keywords:** Glottic insufficiency, Vocal fold atrophy, Sulcus, Bilateral medialization thyroplasty, Type 1 thyroplasty, Laryngeal framework surgery

## Abstract

**Purpose:**

To evaluate voice outcome after bilateral medialization thyroplasty in patients with non-paralytic glottic insufficiency due to vocal fold atrophy with or without sulcus.

**Methods:**

Retrospective cohort study on 29 patients undergoing bilateral medialization thyroplasty for vocal fold atrophy (14 procedures) or atrophy with sulcus (15 procedures) between October 2012 and November 2017. Voice data were collected and analyzed for the preoperative and the 3- and 12-month postoperative time point according to a standardized protocol, including Voice Handicap Index (VHI)-30 and perceptual, acoustic and aerodynamic parameters. Failure rate was based on number of revisions within 12 months and non-relevant improvement (< 10 points) in VHI-30 at 12 months.

**Results:**

There was a clinically relevant (≥ 15 points) and statistically significant improvement (*p* < 0.0001) in the VHI-30 (preoperative: 55.8 points; postoperative at 12 months: 30.9 points). Fundamental frequency for male subjects decreased significantly from 175 to 159 Hz (*p* = 0.0001). The pre- and post-operative grade of dysphonia was significantly lower in patients with atrophy compared to atrophy and sulcus (mean difference 0.70, *p* = 0.017).

**Conclusion:**

Bilateral medialization thyroplasty is a valid treatment option for patients with atrophy with or without sulcus. Outcomes are comparable to other methods reported in literature. However, there is a great need for larger, prospective studies with long-term follow-up to gain more insight into the comparative voice outcomes for the different forms of surgery for patients with glottic incompetence due to atrophy with or without sulcus.

## Introduction

Non-paralytic glottic insufficiency is a common cause of dysphonia affecting both voice quality as vocal function and causing substantial patient’s burden. There are several underlying etiologies, including hypomobility, paresis and vocal fold atrophy. In our practice we distinguish three forms of vocal fold atrophy: (1) vocal fold atrophy in presbyphonia, (2) an adolescent form of vocal fold atrophy, and (3) atrophy associated with congenital vocal fold scar in the form of sulcus [1, 2]. In our patient population pathological sulcus is defined as type II and III according to Ford, and/or sulcus vergeture and sulcus vocalis according to Bouchayer and Cornut [3, 4].

The main surgical treatment for atrophy is vocal fold medialization. This can be achieved by vocal fold injection (VFI) with short acting injectables such as hyaluronic acid, or with durable injectables such as autologous fat or calcium hydroxyapatite. Alternatively, medialization can be accomplished by bilateral medialization thyroplasty. For the treatment of atrophy associated with sulcus epithelium freeing techniques can be used as an alternative or in addition to medialization [5]. At this moment there is no evidence-based decision algorithm available to identify the optimal treatment for an individual patient with sulcus [6]. In their consensus report, the European Laryngological Society (ELS) has suggested medialization as the initial treatment, as it is the least traumatizing to the vocal fold [5]. Several studies have however shown the results of medialization to be less predictable for patients with atrophy associated with sulcus or vocal fold scar compared to those for patients with hypomobility/paresis and atrophy alone. This is thought to be due to the “double pathology” affecting both glottic closure and vibratory potential of the vocal fold in patients with sulcus or vocal fold scar [7].

In a previous study we showed that VFI with autologous fat resulted in equivalent subjective voice improvements for up to 12 months in patients with atrophy and patients with atrophy associated with sulcus [2]. In this study, we evaluated the results after bilateral medialization thyroplasty in patients with vocal fold atrophy with or without sulcus. The aim of this and previous studies on this topic is to contribute to the ongoing attempts to identify the optimal, evidence-based treatment for an individual patient with atrophy or atrophy with sulcus.

## Methods

### Patients

**Fig. 1:**
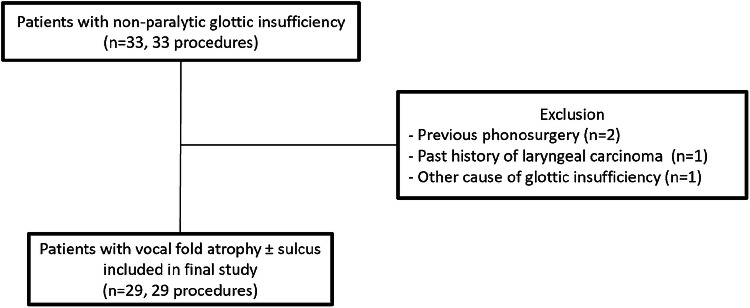
Patient selection and inclusion and exclusion criteria

All patients with non-paralytic glottic insufficiency who underwent bilateral medialization thyroplasty under local anaesthesia (*n* = 33) from December 2011 to November 2017 were retrospectively reviewed. Four patients were excluded because of previous phonosurgery (*n* = 2), past history of laryngeal carcinoma (*n* = 1), or another cause of glottic insufficiency (bilateral paresis, *n* = 1). All of the 29 remaining patients had a complete Voice Handicap Index (VHI)-30 questionnaire pre- and post-operatively and were included in the definitive analysis (Fig. 1). These patients had undergone bilateral medialization thyroplasty between October 2012 and November 2017. The study was approved by Leiden University Medical Center Ethics Committee.

### Voice data

Voice outcome data were collected according to a standardized voice analysis protocol implemented preoperatively and at 3 and 12 months postoperatively. This protocol including patients’ self-assessments using the VHI-30, perceptual evaluation by experienced raters using the overall grade score (*G*) of the GRBAS (Grade, Roughness, Breathiness, Asthenia, Strain) scale, aerodynamic evaluation with maximum phonation time (MPT) and dynamic range, and acoustic analyses including fundamental frequency (F0) and melodic range, has been extensively described in previous publications [1, 2]. A score of 15 points or more in VHI-30 was used to identify patients with voice problems in daily life [8, 9]. A change in pre- and post-operative score of 10 points or more in the individual patient and 15 points or more for a group was considered clinically relevant [9].

### Procedure

All procedures were performed by an experienced laryngologist. Bilateral medialization thyroplasty with Gore-tex^®^ (GORE-TEX^®^ Soft Tissue Patch, Gore Medical, Flagstaff, Arizona) was performed under local anaesthesia in the operating room. The operation technique used was as described by Isshiki and McCulloch with some modifications [10, 11]; starting with a horizontal skin incision at the level of the thyroid, raising subplatysmal flaps, dividing the strap muscles in the midline and visualizing the thyroid alas. The outer perichondrium was incised and a superiorly based flap was raised. The position and the size of the first cartilage window was determined. The window was shaped using a drill and the Kerrison ronguer. The position of the window was checked endoscopically and a similarly positioned window was made in the other ala. Using a 4 mm Gore-tex^®^ patch, cut as a ribbon, optimal medialization was achieved based on three factors: the perceptive evaluation of the voice by the patient and the surgical team, the ease of phonation as evaluated by the patient and the visual evaluation of vocal fold closure and vibration using intraoperative videolaryngostroboscopic guidance. The implant was stabilized with a fixation plate. The wound was closed in layers, leaving a drain in situ. All patients had absolute voice rest the first four days postoperatively. Subsequently, they received speech therapy by an experienced speech-language therapist, starting in the second postoperative week. This included resonant voice therapy and vocal hygiene advice.

### Statistical analysis

All data were analyzed using SPSS (IBM SPSS Statistics for Windows, Version 21.0, released 2012. IBM Corp, Armonk, NY, USA). Demographic details were presented as the mean with standard deviation (SD) or as proportions using percentages. The effect of time on the different voice parameters was assessed with the linear mixed model and was adjusted for diagnosis (atrophy versus atrophy with sulcus). For all statistical tests, a *p* value < 0.05 was considered significant.

## Results

Table 1 shows the preoperative demographic details of the 29 patients; 14 patients had atrophy and 15 patients had atrophy with sulcus.

**Table 1:** Demographic details of the patients undergoing bilateral medialization thyroplasty

Characteristics	Total = 29 (100%)
Mean age, years at baseline (SD)	50.5 (17.9)
Gender, *n* (%)	
Male	12 (41.4)
Female	17 (58.6)
Aetiology, *n* (%)	
Atrophy	14 (48.3)
Atrophy with sulcus	15 (51.7)

*SD* standard deviation

Table 2 shows the changes in voice parameters for the overall patient group. The mean VHI-30 score improved from 55.8 to 30.9 in 12 months (Δ24.9), which was a clinically relevant and a statistically significant improvement (*p* < 0.0001). Fundamental frequency for males decreased from 175 to 159 Hz (*p* = 0.0001) (normal value male: 100–130 Hz). Postoperative changes in other voice parameters were not significant.

**Table 2:** Pre- and post-operative voice outcome data of patients with vocal fold atrophy ± sulcus undergoing bilateral medialization thyroplasty

	Preoperative	3 months postoperative	12 months postoperative	*p* value
	Mean (95% CI)	Mean (95% CI)	Mean (95% CI)	
VHI-30	55.8 (48.4–s63.1)	33.0 (25.6–40.3)	30.9 (22.8–38.9)	< 0.0001*
Grade	1.9 (1.6–2.3)	1.6 (1.3–1.9)	1.7 (1.4–2.1)	0.059
MPT (sec)	11.3 (9.2–13.3)	12.8 (10.8–14.9)	12.6 (10.5–14.8)	0.221
Dynamic range (dB)	30.2 (25.3–35.2)	36.2 (31.4–41.1)	32.3 (27.1–37.5)	0.065
F0 male (Hz)	175 (137–213)	152 (114–190)	159 (121–198)	0.0001*
F0 female (Hz)	224 (167–252)	200 (172–228)	221 (191–251)	0.284
Melodic range (ST)	17.8 (14.9–20.7)	17.7 (14.8–20.5)	18.7 (15.6–21.8)	0.784

^*^p value < 0.05 was considered significant

*CI *confidence interval, *VHI* Voice Handicap Index, *MPT* maximum phonation time, *F0* fundamental frequency, Hz hertz, *dB* decibels, *ST* semitones

Table 3 shows the results stratified for patients with vocal fold atrophy versus patients with vocal fold atrophy and sulcus. At baseline the pre-operative values of the different voice parameters were comparable between the patient groups except for grade of dysphonia. VHI-30 showed significant improvement for both patient groups and this improvement was also clinically relevant in both groups (Δ29.9 atrophy; Δ19.0 sulcus). The perceptive rating of the grade of dysphonia showed no significant improvement, but was significantly lower both pre- and postoperatively in patients with atrophy, with a grade corresponding to mild dysphonia (1.6–1.4) compared to patients with atrophy and sulcus, with a grade corresponding to moderate dysphonia (2.3–2.1) (mean difference 0.70, *p* = 0.017). Dynamic range in the atrophy group showed a significant improvement at three months, but this effect had disappeared at 12 months. Finally, fundamental frequency for males in both groups showed significant lowering postoperatively (atrophy *p* = 0.001, sulcus *p* = 0.015).

**Table 3:** Pre- and post-operative voice outcome data stratified for patients with vocal fold atrophy and vocal fold atrophy with sulcus

Aetiology	Preoperative	3 months postoperative	12 months postoperative	*p* value
	Mean	Mean	Mean	
VHI-30				
Atrophy	56.9	29.9	27.0	< 0.0001*
Sulcus	54.4	34.9	35.4	0.002*
Grade				
Atrophy	1.6	1.3	1.4	0.284
Sulcus	2.3	1.9	2.1	0.204
MPT (s)				
Atrophy	11.5	13.7	13.6	0.221
Sulcus	11.0	12.0	11.7	0.769
Dynamic range (dB)				
Atrophy	29.0	38.6	31.1	0.028*
Sulcus	31.4	34.1	33.4	0.732
F0 male (Hz)				
Atrophy	160	133	139	0.001*
Sulcus	190	172	179	0.015*
F0 female (Hz)				
Atrophy	234	197	212	0.263
Sulcus	215	203	230	0.548
Melodic range (ST)				
Atrophy	17.1	19.5	19.6	0.446
Sulcus	18.5	16.0	17.9	0.482

^*^p value < 0.05 was considered significant

*VHI* Voice Handicap Index, *MPT* maximum phonation time, *F0* fundamental frequency, *Hz* hertz, *dB* decibels, *ST* semitones

All 29 procedures had complete follow-up at 3 months and 24 procedures had complete follow-up at 12 months. Five procedures did not have 12-month data: three patients (*n* = 3) had already undergone or opted for a revision thyroplasty and 2 patients (*n* = 2) were lost to follow-up. Out of the 24 procedures of which voice outcome at 12 months could be calculated, 19 had a clinically relevant improvement (≥ 10points) in VHI-30. Five out of 24 had a non-relevant improvement (< 10 points). One of the patients with non-relevant improvement opted to undergo revision thyroplasty at the 12-month visit, bringing the total number of revisions to four.

We estimate the failure rate within 12 months for individual procedures to be somewhere between 13.8% (4/29 procedures) if considering only procedures requiring revision thyroplasty as failures and 27.6%% (8/29 procedures) if considering procedures requiring revision thyroplasty and procedures without clinically relevant VHI-improvement at 12 months as failures. If, additionally, the two procedures lost to follow-up are included as failures, the rate would be 34.5%. Looking at the revisions in more detail the VHI significantly improved at 12 months after revision in two patients, one with atrophy and one with sulcus. In two patients, both with sulcus, the VHI 12 months after revision was similar to the values before the primary intervention.

## Discussion

In this study, we present our results after bilateral medialization thyroplasty in patients with atrophy with or without sulcus. We found a statistically significant and clinically relevant subjective improvement in both patient groups. Based on this, we conclude that bilateral medialization thyroplasty is an effective treatment option for both atrophy and atrophy with sulcus. Although VHI-scores improved significantly they remained above normal limits, implying a diminished but ongoing voice burden for these patients after surgery. It is important to incorporate this information in patient counselling.

As stated, VHI outcome was similar for patients with atrophy with or without sulcus. In the perceptual grading and in dynamic range there were some differences between the two groups. Postoperatively, patients with atrophy only had an overall lower grade score, which corresponded to mild dysphonia, compared to patients with atrophy and sulcus, which corresponded to moderate dysphonia. This difference was already present pre-operatively and could be well explained by the more complex “double pathology” in the latter group, resulting in a more severe perceptual dysphonia. For dynamic range the results showed a significant improvement at 3-months postoperatively in patients with atrophy, but this effect had dissolved at 12 months. We have no solid explanation for this temporary improvement in dynamic range. It may be related to characteristics of the Gore-Tex and could possibly be explained by the malleability of the material. In time, this could cause it to change shape, position or volume. The fact that this increase was not seen in the patients with sulcus could be because of the earlier mentioned “double pathology”. Lastly, we found a lowered, but still higher than normal fundamental frequency in males after bilateral medialization thyroplasty, both for atrophy and atrophy with sulcus. This may be an interesting aspect for further research as gender specific outcomes after medialization thyroplasty for non-paralytic glottic incompetence has gained more attention recently, showing female having greater subjective improvement compared to male after surgery [12]. We would also consider to include this aspect in the pre-operative counselling of male patients by informing them that their voices may still be higher pitched after surgery.

Thyroplasty as surgical treatment for vocal fold atrophy, with or without sulcus, became more popular after Isshiki et al. reported promising results in the mid-1990′s [13]. Shortly after this, Gore-tex^®^ medialization thyroplasty was introduced [11, 14]. Since then several studies have reported on bilateral medialization thyroplasty in patients with glottic incompetence as a result of atrophy and sulcus, as well as paralysis, paresis, and other causes [6, 7, 14–23]. The largest patient group (total *n* = 75, hypomobility *n* = 27, paresis *n* = 16, scarring *n* = 18, atrophy *n* = 14), which also has the longest follow-up (up to 10 years), showed overall improvement in voice-related quality of life (VRQOL). This improvement was statistically significant in most subgroups. Notably however, for the scar subgroup there was no statistically significant improvement in VRQOL [7].

Only a few additional studies have reported specifically on bilateral medialization thyroplasty in patients with atrophy or atrophy with sulcus/scar [6, 18, 20]. Sachs et al. showed significant improvement in self-rating measures [VRQOL, Glottal Function Index (GFI) and a “best voice” Visual Analogue Scale (VAS)] in their patients with age-related atrophy treated with bilateral medialization thyroplasty [18]. Allensworth et al. found a significant improvement in the VHI-30 (59.4–31.5) in 10 patients, which was very similar to the improvement found in our study (55.8–30.9) [20]. Contrary to our study where we only found an impact on fundamental frequency (males) and a transient effect in the dynamic range (patients with atrophy only), both Sachs and Allensworth showed improvements in several of the (semi-)objective parameters they analyzed [18, 20]. As for patients with sulcus/scar, Welham et al. showed a significant improvement in VHI-30 after treatment with thyroplasty in his series which did include unilateral procedures. The median VHI-30 in thyroplasty group improved from 59.0 to 39.3 (average post-treatment value at 18 months) or 31.0 (best post-treatment value in 18 months) (personal communication with author) [6].

Several studies have reported less promising results after medialization thyroplasty for patients with scar compared to other causes of non-paralytic glottic incompetence in the past and also more recently [7, 13, 17, 21]. Although we did anticipate more negative results for our sulcus patients, we did not see this in our own series [1, 2], including this study. This may be due to our inclusion criteria, as we have only included sulcus and not iatrogenic scar. From assessing the revision thyroplasties in this study (*n* = 4), which occurred in both atrophy and atrophy with sulcus patients, we concluded that there are probably different reasons for disappointing results. Some failures may be due to an inadequate procedure, such as too little or too much medialization, which can be corrected, but that some failures are also likely due to the inherent limitations of this technique in treating this patient cohort.

Concerning the duration of the effect of thyroplasty, we did not have data beyond twelve months. However, Domingues et al. showed that the results of medialization thyroplasty in her patients with non-paralytic glottic incompetence were stable at a median follow-up of 16.3 months [19]. We conclude from the above that there is evidence in literature to support our finding that bilateral medialization thyroplasty provides long-term, subjective improvement in patients with vocal fold atrophy and atrophy with sulcus. The relationship between the relatively constant improvement in subjective parameters, and the varying improvement in the multitude of the (semi-)objective parameters studied so far is still not clear [1]. With “(semi-)objective” parameters we mean all stroboscopic, aerodynamic and acoustic voice outcome measurements described in the literature. We added “semi-” because these measurements are not entirely objective, as patient performing factors and investigator interpretation factors may influence the outcome. If (semi-)objective voice parameters should be used, and if so, which parameters are suitable, needs further investigation.

Several studies have compared medialization thyroplasty in patients with glottic incompetence to other treatments modalities such as VFI medialization and alternatively microphonosurgery in the case of sulcus/scar. In their earlier mentioned study, Dominguez et al. compared medialization thyroplasty (*n* = 20) to VFI with autologous fat injections (*n* = 15) in patients with atrophy, although their series did include patients with paresis [19]. It showed improved subjective voice outcome with clinically relevant and statistically significant VHI-10 improvements for both techniques; even showing a normalization of the VHI-10 in thyroplasty patients at 7 months (decrease from 30.5 to 9.1; normal value VHI-10 English version ≤ 11 [24]). Moreover, as stated earlier, the thyroplasty group maintained this effect during the whole follow-up period in contrast to the VFI group, in which values had regressed to pretreatment scores at around 16 months. In accordance with Dominguez, we also found the subjective outcome of our bilateral thyroplasty patients to be comparable to a similar cohort of our patients treated with bilateral VFI with autologous fat [2]. VHI-30 scores for our VFI patients decreased from 49.1 (pretreatment) to 27.9 (12 months post-treatment) which is very similar to VHI-30 outcomes in our present study. In a prospective trial performed by Welham et al., comparing three types of surgery for sulcus [type I thyroplasty (*n* = 9), injection laryngoplasty (*n* = 9), and graft implantation (*n* = 10)] both thyroplasty and graft implantation yielded significant improvement in VHI-30, although graft implantation only showed significant VHI-improvement in best post-treatment score and had a slow and long recovery period. No significant changes were found in perceptual, acoustic, aerodynamic and vocal fold physiology outcomes. Although subjective improvement after thyroplasty was clinically relevant and statistically significant, authors advised caution in their conclusions, because of the wide variation in response seen across individual patients and different treatment groups [6].

Finally, several studies have published results for microphonosurgical techniques in treating sulcus/scar [25–38]. Techniques vary and include: microflap with or without prosthesis (fibrin glue, fascia, fat), slicing techniques, superficial steroid injections and fat injections as well as laser procedures with PDL and KTP lasers. The mean decrease in VHI-30 in the publications from which this could be extracted [27, 29, 33, 36–38]; ranges from 1.7 [29] to 30.6 [38] points with an average decrease of 20.1 points. Although this does not constitute a qualified systematic review or meta-analysis, when comparing it to the average VHI-30 decrease in this study (24.9 points) it gives a rough indication that bilateral medialization thyroplasty may be as effective in treating sulcus/scar as performing microphonosurgery.

There were some limitations of this study. One was the retrospective study design. Although missing data were limited and corrected for, the retrospective study design implied selection bias. Other limitations were the size of the subgroups of our patient population and the limited follow-up time of 12 months, as already mentioned above.

## Conclusion

This study together with a review on this topic show that bilateral medialization thyroplasty is a valid treatment option for patients with atrophy with or without sulcus. Outcomes are comparable to other methods although there are indications that they may be more stable than in VFI medialization. In the case of sulcus/scar results are reached more quickly, and potentially with less risk of permanent deterioration than in microphonosurgery. However, there is a great need for larger, prospective studies with long-term follow-up to gain more insight into the comparative voice outcomes for the different forms of surgery for patients with glottic incompetence due to atrophy with or without sulcus. Also, more data are needed on which subjective and (semi-)objective voice parameters are reliable and meaningful in the evaluation of these outcomes.
